# Murine model of acute myocarditis and cerebral cortical neuron edema induced by coxsackievirus B4

**DOI:** 10.24272/j.issn.2095-8137.2017.056

**Published:** 2018-02-09

**Authors:** Zhao-Peng Dong, Qian Wang, Zhen-Jie Zhang, Michael J. Carr, Dong Li, Wei-Feng Shi

**Affiliations:** 1 Key Laboratory of Etiology and Epidemiology of Emerging Infectious Diseases in Universities of Shandong, Taishan Medical University, Taian Shandong 271000, China; 2 Shanghai Jinshan Center for Disease Control and Prevention, Shanghai 201599, China; 3 School of Public Health, Taishan Medical University, Taian Shandong 271016, China; 4 Global Station for Zoonosis Control, Global Institution for Collaborative Research and Education (GI-CoRE), Hokkaido University, Sapporo 060-8589, Japan; 5 National Virus Reference Laboratory, School of Medicine, University College Dublin, Dublin 4, Ireland

**Keywords:** Coxsackievirus B4, Myocarditis, CNS, Edema, Neurons

## Abstract

Globally, coxsackievirus B4 (CV-B4) has been continuously isolated and evidence suggests an association with the development of pancreatitis and type I diabetes. In addition, CV-B4 is also associated with myocarditis and severe central nervous system (CNS) complications, which remain poorly studied and understood. In the present study, we established an Institute for Cancer Research (ICR) mouse model of CV-B4 infection and examined whether CV-B4 infection resulted in a predisposition to myocarditis and CNS infection. We found high survival in both the treatment and control group, with no significant differences in clinical outcomes observed. However, pathological lesions were evident in both brain and heart tissue of the CV-B4-infected mice. In addition, high viral loads were found in the neural and cardiac tissues as early as 2 days post infection. Expressions of IFN-γ and IL-6 in sera were significantly higher in CV-B4-infected mice compared to uninfected negative controls, suggesting the involvement of these cytokines in the development of histopathological lesions. Our murine model successfully reproduced the acute myocarditis and cerebral cortical neuron edema induced by CV-B4, and may be useful for the evaluation of vaccine candidates and potential antivirals against CV-B4 infection.

## INTRODUCTION

Coxsackievirus B (CV-B) comprises a number of established cardiomyopathy-associated viral serotypes, taxonomically classified within the genus *Enterovirus* species B, family *Picornaviridae*. There are currently 63 recognized enterovirus serotypes (www.picornaviridae.com) with diverse tissue tropisms and correspondingly broad spectrum of disease presentation, including myocarditis, encephalitis, paralysis, meningitis, upper and lower respiratory disease, pleurodynia, herpangina, myopericarditis, pancreatitis, and type I diabetes (Knowles et al., 2011; Tapparel et al., 2013). In the prior two decades, the relationship between CV-B and myocarditis/dilated cardiomyopathy has been substantiated. For example, CV-B infections have been identified in approximately 25%–40% cases of acute myocarditis and dilated cardiomyopathy in young adolescents and adults (Gaaloul et al., 2014; Leonard, 2004). Furthermore, an epidemiological survey has revealed the co-circulation of six serotypes of CV-B in a single location in Changchun, Jilin Province, China, with the predominant agents of viral myocarditis in infants from 2002–2011 being CV-B3 (30.1%–36.5%), CV-B4 (18.0%–24.8%), and CV-B5 (3.3%–8.1%) (Zhang et al., 2014). Significantly, CV-B4 is also associated with severe central nervous system (CNS) complications and neurological sequelae (Zhu et al., 2015).

Previous research has employed mouse models to study CV-B4 infection. These studies have mainly focused on the inflammatory insults in exocrine tissue damage (De Palma et al., 2009) and the association with type 1 diabetes (McCall et al., 2015) during CV-B4 infection. Due to the lack of a stable mouse model of CV-B4 infection, the exact mechanism(s) of how CV-B4 results in myocarditis and CNS invasion remain unclear. Furthermore, pathogenesis studies are impeded by the absence of appropriate animal models of disease that would facilitate the development of prophylactic vaccines and antivirals for the prevention and treatment of acute disease.

We investigated whether CV-B4 infection results in a predisposition to myocarditis and CNS infection in Institute for Cancer Research (ICR) neonatal mice. Dynamic detection of viral loads, hematoxylin and eosin staining, and immunohistochemical examination were performed to determine tissue tropisms, pathological lesions, and distribution of CV-B4 *in vivo*, respectively. Finally, the expression levels of virus-induced inflammatory cytokines were examined and correlated with viral titers and clinical scores.

## MATERIALS AND METHODS

### Virus and cells

Human laryngeal carcinoma epithelium (Hep-2) cells were cultured in Dulbecco's modified Eagle's medium (DMEM; Gibco, USA), supplemented with 10% fetal bovine serum (Gibco) and 1% penicillin-streptomycin at 37 °C under a 5% CO_2_ atmosphere. The CV-B4 LY114F strain employed in the present study was isolated from the stool of a three-year-old patient who presented with classical symptoms of hand, foot, and mouth disease (HFMD) in Linyi city, Shandong Province, China, in 2015. Virus propagation in Hep-2 cells and 50% tissue culture infective doses (TCID_50_) were determined in accordance with the methods of Reed & Muench (1938).

### Ethics statement and animal infection model

Institute for Cancer Research (ICR) mice were purchased from Beijing Vital River Laboratory Animal Technology Co., Ltd. The animal experiments were approved by the Taishan Medical College Administrative Committee for Laboratory Animals, and all procedures involving animals were performed in accordance with the Shandong Laboratory Animal Welfare and Ethics Administrative Committee.

Three-day-old ICR mice were challenged with the CV-B4 LY114F strain (10^7^ TCID_50_/animal) by intramuscular (i.m.) injection, with control groups injected with DMEM. Body weights, clinical manifestations, and survival rates of mice infected with LY114F were monitored daily until 10 days post infection (dpi). Multivariate analysis was used to analyze the differences in body weights between infected mice and negative controls at each observation time point. Clinical criteria were scored as follows: 0, healthy; 1, lethargy and inactivity; 2, hind limb weakness; 3, single limb paralysis; 4, double hind limb paralysis; and 5, death or dying.

### Dynamics of CV-B4 viral RNA titers in infected mice

Brain, heart, contralateral hind limb skeletal muscle, lung, intestine, spleen, and blood samples from each mouse (*n*=3 per time point) infected with LY114F were collected at 1–5 dpi, respectively. Viral RNA was extracted from equivalent weights of tissue samples and volumes of blood samples from infected and control mice with TRIzol reagent (Takara, China), as per the manufacturer’s instructions. cDNA was generated using a reverse transcription kit (Takara, China) for 45 min at 42 °C and a GoldStar TaqMan Mixture kit (CWBIO) was employed for real-time PCR. Oligonucleotide primers and hydrolysis probe sequences are as follows: sense, 5'-CCTGAATGCGGCTAATCC-3'; antisense, 5'-TTGTCACCATWAGCAGYCA-3'; and hydrolysis probe, 5'-FAM-CCGACTACTTTGGGWGTCCGTGT-BHQ1-3'. Real-time PCR thermocycling was performed on a LightCycler 96 platform with the following conditions: 95 °C for 10 min; 40 cycles of 95 °C for 15 s, and 60 °C for 1 min. Standard curves for absolute quantification of CV-B4 copy numbers in different tissue and blood samples were established, as described previously (Zhang et al., 2017a, b).

### ELISA measurement of cytokine levels in sera

Peripheral blood was collected from 3-day-old mock-infected controls or mice infected with a 10^7^ TCID_50_/animal dose of LY114F from 1–5 dpi (*n*=3 per time point). Peripheral blood was centrifuged immediately at 10 000 r/min for 10 min at room temperature and sera were stored at –80 °C prior to use. The levels of IFN-γ, IL-6, IL-4, IL-1β, IL-10, IL-13, IL-18, and TNF-α in sera were determined by commercial ELISA kits (Multisciences Biotechnology, China), according to the manufacturer’s protocols.

### Histopathological and immunohistochemical staining

Three-day-old mice were infected with LY114F (10^7^ TCID_50_/animal). Brains, hearts, muscles, and lungs from experimental mice and controls were subjected to histopathological and immunohistochemical examination. Tissues were fixed in 10% neutral buffered formalin for 72 h and then embedded in paraffin, as per previously described procedures (Yu et al., 2014). For pathological examinations, formalin-fixed, paraffin-embedded sections (4 μm thick) stained with hematoxylin and eosin were employed. For immunohistochemical examination, paraffin-embedded sections were dewaxed, dehydrated, and microwaved for 15 min at 95 °C to 99 °C in EDTA buffer (1 mmol/L). Polyclonal mouse anti-CV-B4 antibody (1:300 dilution; Abcam, UK) was applied for 15 h at 4 °C. A secondary horseradish peroxidase-conjugated goat anti-mouse IgG (1:400 dilution; ZSGB-BIO, China) was then applied for 30 min at room temperature, followed by avidin-biotin-peroxidase complex and 3,3'-diaminobenzidine tetrahydrochloride chromogen (1:1000 dilution; ZSGB-BIO, China). Subsequently, tissue sections were counterstained with hematoxylin and negative controls were incubated with PBS instead of primary antibody.

### Statistical analysis

All statistical analyses were performed with the Statistical Analysis System 9.2 (SAS, USA). Differences in mean tissue viral load and serum cytokine concentrations were determined with two-tailed analysis of variance (ANOVA). A difference was considered significant at *P*<0.05.

## RESULTS

### Establishment of the CV-B4 infection mouse model

**Figure 1:**
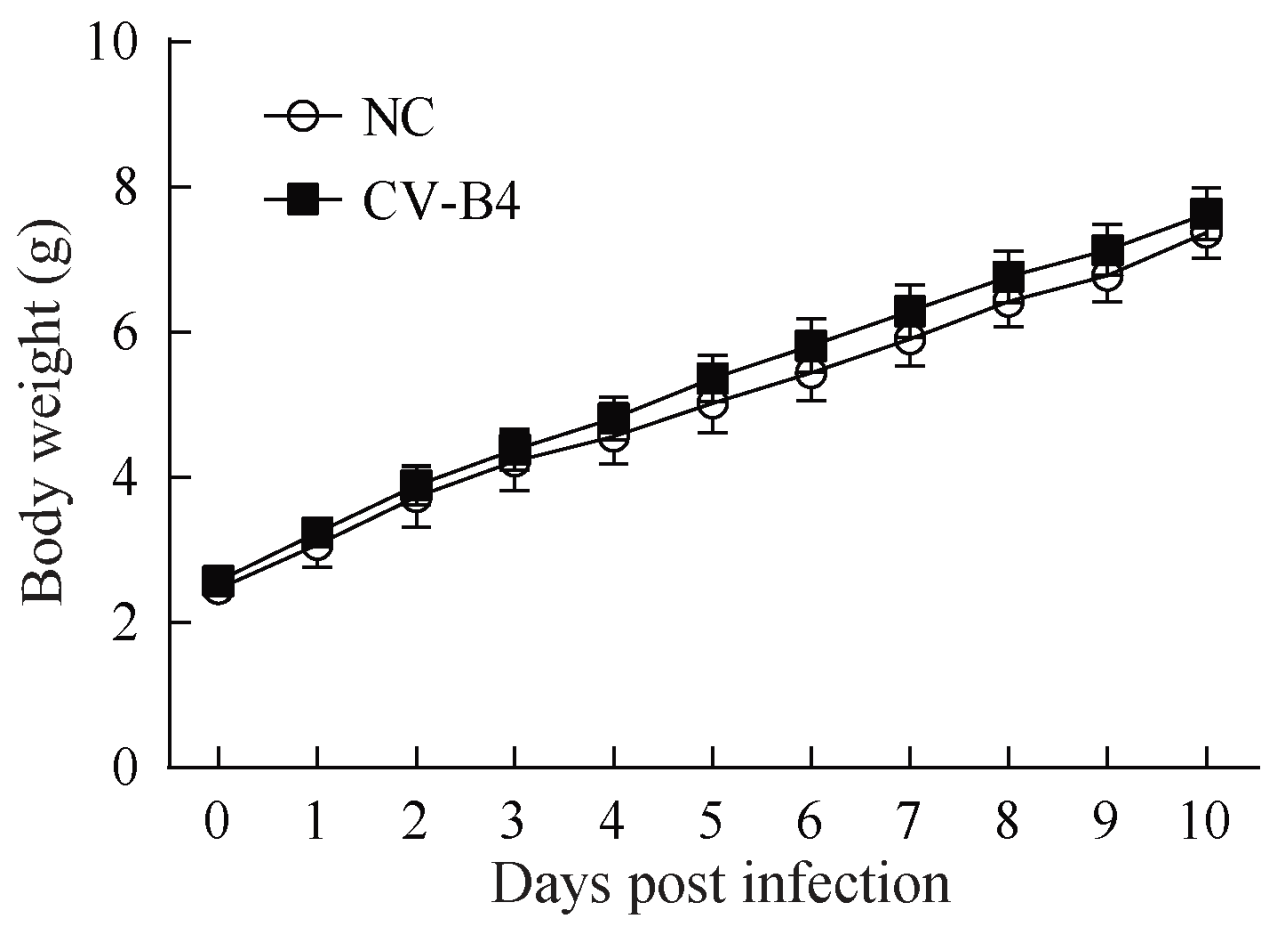
Body weights of ICR mice infected with the CV-B4 LY114F strain

Multivariate analysis showed that the body weights of the infected mice were significantly higher than those of the negative controls at 6 dpi (*F*=5.439, *P*=0.031), 8 dpi (*F*=9.521, *P*=0.008), 9 dpi (*F*=8.526, *P*=0.009), and 10 dpi (*F*=10.601, *P*=0.004) (Figure 1). There were no significant differences between the CV-B4 LY114F-treated mice and the control group with respect to mean clinical score, and survival rate.

Control animals were inoculated with saline. All mice were monitored daily for body weight until 10 dpi.

### Histopathological and immunohistochemical examination

**Figure 2:**
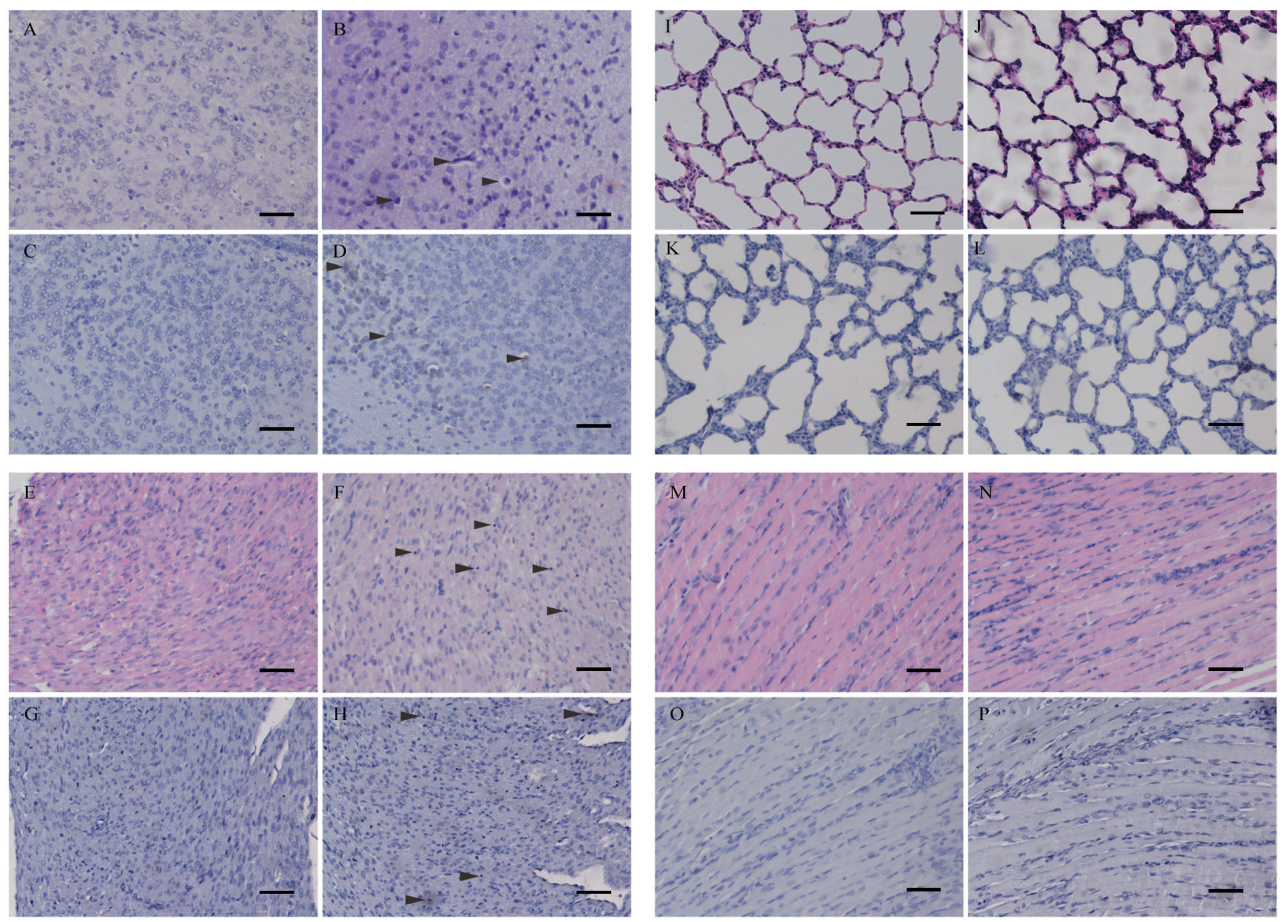
HE and IHC analyses of infected 3-day-old mice after i.m. challenge with the CV-B4 LY114F strain

To examine the histopathological lesions and distribution of viral antigens in mice post infection, histopathological analysis and immunohistochemical examination of the brains, hearts, skeletal muscles, and lungs were performed. Results showed evident pathological lesions in the brain (Figure 2A–D) and heart (Figure 2E–H) tissues in mice infected with CV-B4 LY114F, including cerebral cortical neuron edema (Figure 2B) and myocardial lymphocytic infiltration (Figure 2F). Furthermore, immunohistochemical analysis demonstrated the presence of CV-B4 antigens in brain neurons (Figure 2D) and myocardial fibers (Figure 2H); however, there were no distinct histopathological lesions or antigen detectable in the lung (Figure 2I–L) or skeletal muscle tissues (Figure 2M–P).

No histological changes were observed in the brain, heart, lung, or hind limb muscle tissue of the negative control mice (A, E, I, and M), as well as in the lung (J) or muscle (N) tissues of the infected mice. Infected mice exhibited cerebral cortical neuron edema in the brain (B) and myocardial lymphocytic infiltration in heart tissue sections (F), as indicated by arrows. IHC analyses showed the existence of viral antigen in the brain and heart tissue of infected mice (D, H). There was no antigen detected in the lung (L) and muscle (P) tissue of the infected mice and in the mice challenged with the PBS buffer (C, G, K, and O). Magnification: ×200, scale bars: 100 μm.

### Dynamics of CV-B4 viral RNA titers in the CV-B4 murine model

**Figure 3:**
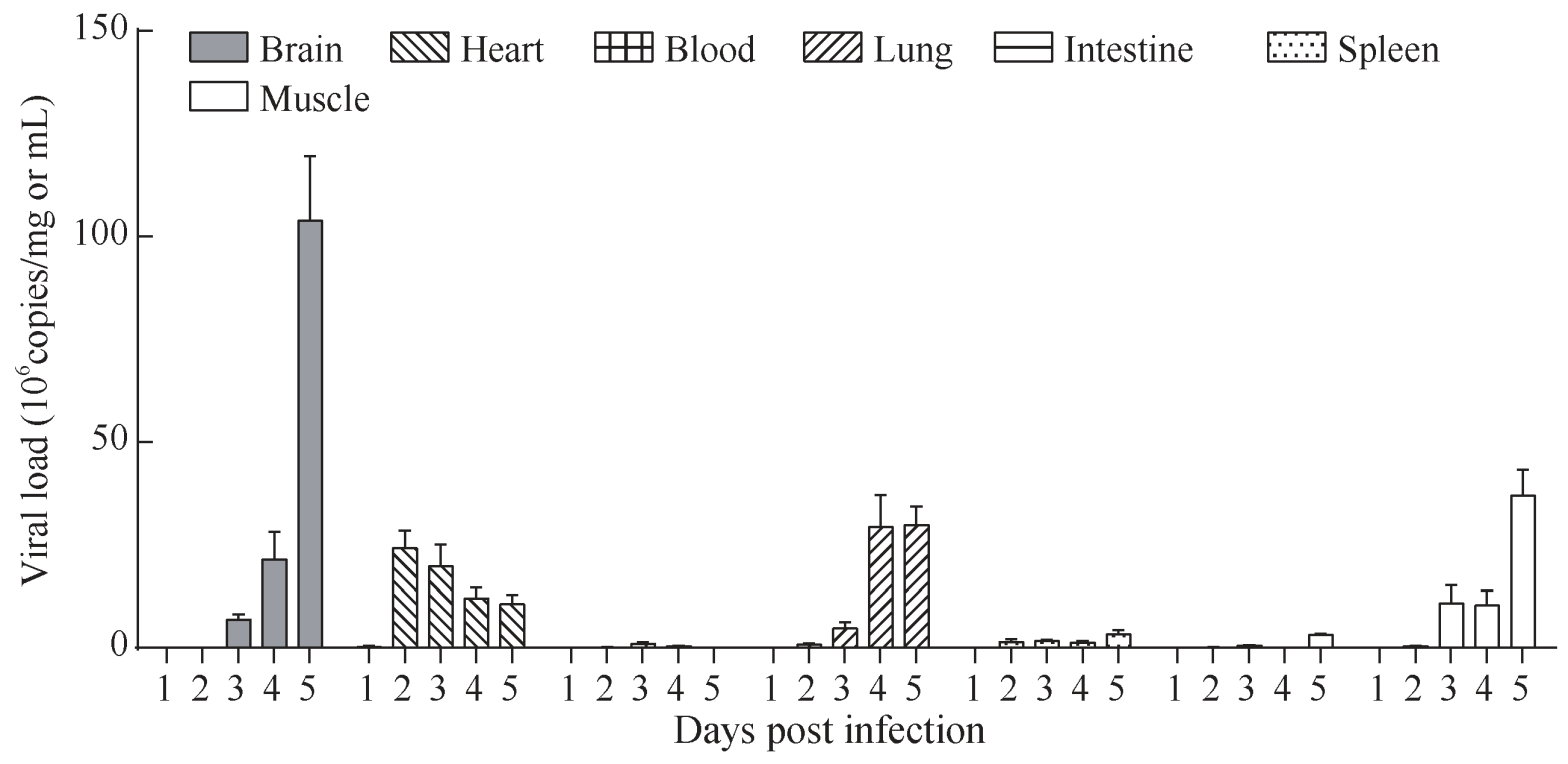
Mean viral loads in tissue and peripheral blood of CV-B4-infected mice

Viral loads in 3-day-old mice inoculated with 10^7^ TCID_50_ of CV-B4 LY114F were measured at 1–5 dpi. The kinetics of viral loads in the brain, heart, contralateral hind limb skeletal muscle, lung, intestine, spleen, and blood samples are illustrated in Figure 3. In the infected group, CV-B4 RNA was detectable in the brain at 3 dpi and peaked at 5 dpi with a viral load of >10^8^ copies/mg tissue. Notably, viral loads in the heart were the earliest evident of all organs tested (2 dpi) and gradually decreased from 2.4×10^7^ copies/mg (2 dpi) to 1.1×10^7^ copies/ mg (5 dpi). Additionally, there was a slow increase in the viral titer following infection from days 1 to 5 in lung tissues and skeletal muscle, in which viral loads each peaked at approximately 3.0×10^7^ copies/mg at 5 dpi. In contrast, from 1 to 5 dpi, viral titers in the blood (0.0–1.0×10^6^ copies/mL), intestine (0.0–3.1×10^6^ copies/mg), and spleen (0.0–3.3×10^6^ copies/mg) were much lower.

Three-day-old ICR mice were i.m. inoculated with 10^7^ TCID_50_ of the CV-B4 LY114F strain. Viral loads (10^6^ copies/mg or mL) in the brain, heart, blood, lung, intestine, spleen, and muscle of infected mice were quantified by RT-qPCR. Data represent the mean results of three mice ± standard deviation of the mean.

### Expression of inflammatory cytokines in sera

**Figure 4:**
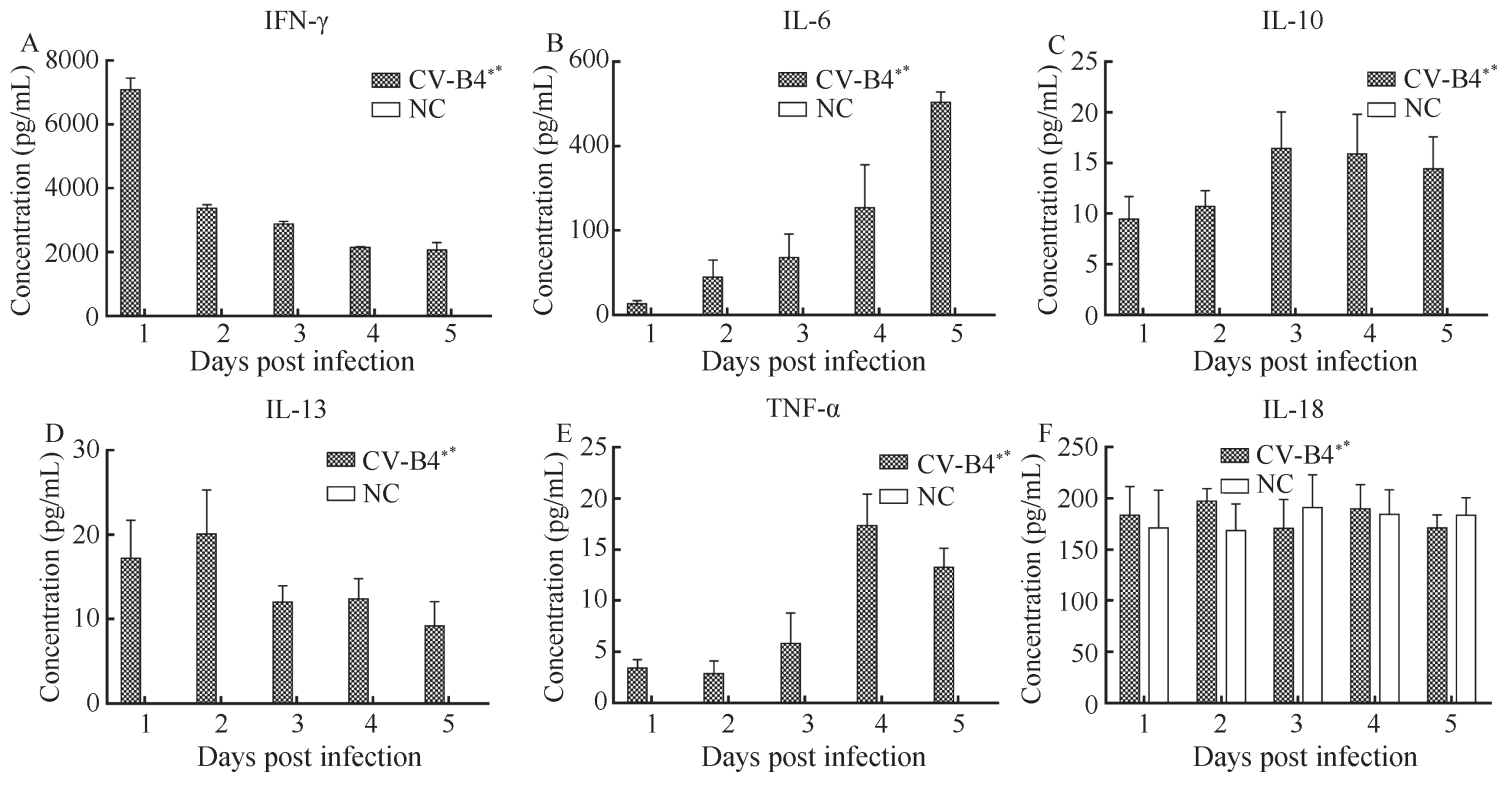
Peripheral blood cytokine expression levels in neonatal mice infected with the CV-B4 LY114F strain

The expressions of inflammatory cytokines, including IFN-γ, IL-6, IL-10, IL-13, TNF-α, IL-18, IL-1β, and IL-4, in sera were measured by ELISA at 1 to 5 dpi in 3-day-old ICR mice infected with CV-B4 LY114F (10^7^ TCID_50_/animal dose). The expressions of IFN-γ and IL-6 in sera were significantly higher in CV-B4-infected mice compared to uninfected negative controls. In CV-B4-infected animals, the expression level of IFN-γ (Figure 4A) in serum peaked very early (~7 100 pg/mL at 1 dpi), and then decreased rapidly to <4 000 pg/mL by 2 dpi; however, high expression levels (>2 000 pg/mL) were maintained up to 5 dpi. In contrast, the expression level of IL-6 (Figure 4B) in serum was low at 1 dpi, but increased steadily during infection and peaked at 5 dpi with a concentration of >500 pg/mL. The titers of IL-10 (Figure 4C) were detectable early in infection (10 pg/mL at 1 and 2 dpi) compared to uninfected controls and peaked at 16.44 pg/mL by 3 dpi, with low expression levels maintained throughout the infection. The titers of IL-13 (Figure 4D) were also detectable early in infection and peaked at 20.11 pg/mL at 2 dpi, before gradually decreasing. The expression of TNF-α (Figure 4E) was lower at earlier time points, and peaked at 17.37 pg/mL by 4 dpi. The titers of IL-18 (Figure 4F) in both experimental and control groups showed high levels of expression (>100 pg/mL) across all time points, and there were no significant differences between the groups. Finally, the expressions of IL-1β and IL-4 were not detectable in either group.

Levels of IFN-γ (A), IL-6 (B), IL-10 (C), IL-13 (D), TNF-α (E), and IL-18 (F) in the sera of 3-day-old ICR mice i.m. inoculated with LY114F and of negative controls (NC) at 1, 2, 3, 4, and 5 dpi were determined using murine ELISA assays. Expression levels of cytokines in sera between experimental and control groups were compared by Fisher’s least significant difference multiple comparison test. *: *P*<0.01. Data represent the mean results of three mice ± standard deviation of the mean.

## DISCUSSION

Previous studies on the CV-B4 viral serotype have primarily focused on cardiotropism, with little emphasis on the pathogenicity of CV-B4 in the CNS (El Hiar et al., 2012; Hu et al., 2012). Earlier reports demonstrated that >90% of reported CV-B infections occur in infants, children, and adolescents (Romero, 2008). Therefore, establishing a sensitive neonatal animal model of CV-B4 infection is important for pathogenesis studies of CV-B4-induced CNS disease in infants. Such models could clarify the pathological processes and enable development of vaccines and antivirals for the prevention and treatment of acute neurological cases.

After 3-day-old neonatal mice were inoculated with 10^7^ TCID_50_ CV-B4 in the present study, viral RNA was detectable in several organs, including brain, heart, lung, and skeletal muscle. Of note, viral titers in the brain were three-fold greater than that in the other organs at the latter stage of infection (5 dpi). In addition, readily discernible pathological changes were detected in both brain and myocardial tissue. Compared with enterovirus species A virus infection models, including EV-A71 (Dong et al., 2016; Yue et al., 2016), CV-A16 (Huang et al., 2015; Mao et al., 2012), CV-A6 (Zhang et al., 2017b), and CV-A10 (Zhang et al., 2017a), which are associated with hind limb paralysis and/or severe respiratory involvement, the enterovirus species B CV-B4 had comparably minor pathology in skeletal muscle and lung tissue.

The interaction between pathogen and the host immune system is critical to develop an understanding of the mechanisms of viral pathogenesis. Clinical studies have shown that the high expressions of several cytokines, such as IL-6, TNF-α, and IL-10, are closely correlated with clinical manifestations and complications associated with CV-B4 infection (Alidjinou et al., 2013; Gu et al., 2009). Our data showed that after neonatal mice were inoculated with CV-B4, there was a gradual increase in the level of IL-6 in serum, different from that observed in enterovirus species A virus infection (CV-A6 and CV-A10) in ICR neonatal mice (Zhang et al., 2017a, b). Our previous studies on CV-A6 and CV-A10 mouse models of infection also showed high expression levels of IL-6 throughout all stages of infection (Zhang et al., 2017a, b). Therefore, we speculate that the differences in pathogenicity between CV-A and CV-B enteroviral species in the same neonatal ICR murine model may be attributable to differences in skeletal muscle tropisms and proinflammatory cytokine expression levels, such as that of IL-6, in peripheral blood. Previous *in vivo* experiments studying EV-A71 infection have shown that treatment with anti-IL-6 neutralizing antibodies increases survival rates, whereas treatment with IL-6 can exacerbate pulmonary dysfunction in EV-A71-infected mice (Khong et al., 2011). We also observed this latter phenomenon of disease exacerbation in CV-A6 and CV-A10 murine models of infection (Zhang et al., 2017a, b).

The coxsackievirus and adenovirus receptor (CAR) is responsible for coxsackie B virus infection in human cells (Bergelson et al., 1997), with non-permissive cells transfected with mCAR cDNA rendered susceptible to CV-B viral infections (Bergelson et al., 1997; Bergelson et al., 1998). CAR is reported to be employed by laboratory reference strains and clinical isolates of all six serotypes of EV-B (Martino et al., 2000); however, different clinical CV-B isolates have been found to possess distinct interactions with CAR (Riabi et al., 2014). In addition, enhanced CAR expression is associated with experimental autoimmune myocarditis in adult mice (Ito et al., 2000) and treatment of CV-B3-infected BALB/c mice with CAR4/7 can aggravate cardiac injury (Dörner et al., 2006). Although CAR expression is significantly higher in dilated cardiomyopathy (DCM) cases than in negative controls, no significant differences in EV viral loads between DCM and non-DCM cases have been observed (Sharma et al., 2016). Consequently, the relationship between increased CAR expression and viral load of enteroviruses, including CV-A and CV-B species, remains unclear and warrants further investigation.

In conclusion, ICR neonatal mice i.m. infected with CV-B4 exhibited significant pathology in the brain and myocardium. This represents a suitable model for the establishment of CV-B4 infection to study the pathogenesis of CNS and cardiac complications and is a resource for the development of both prophylactic vaccines and antivirals to reduce morbidity and mortality in children.
